# FOSTERING COLLABORATION TO BENEFIT OLDER DRIVERS

**DOI:** 10.1093/geroni/igad104.1243

**Published:** 2023-12-21

**Authors:** Laura Hemmy, Nichole Morris, Kyle Shelton

**Affiliations:** University of Minnesota, Minneapolis, Minnesota, United States; University of Minnesota, Minneapolis, Minnesota, United States; University Of Minnesota, Minneapolis, Minnesota, United States

## Abstract

Access to transportation is a fundamental contributor to independence and quality of life, and for many independent driver status is a seminally important part of agency and self-concept. Driving is a lifelong skill utilizing a variety of physical, sensory and cognitive abilities, but also influenced by the community in which one lives, built infrastructure, vehicle design and engineering, social and family networks, and driver insight. Because older drivers are at increased risk for negative crash outcomes, generating knowledge about how to promote safe driving in the older population, when to consider retirement from driving, and compensatory sources of transportation, are of the utmost importance. Faculty and trainees with interest in these questions come from disparate and often unconnected parts of academic research institutions. The University of Minnesota (UMN) Center for Healthy Aging Innovation (CHAI) Special Interest Group (SIG) structure, in collaboration with the UMN Center for Transportation Studies (CTS), has served as a platform for connection. SIG members will share how the opportunity to connect across disciplines, schools and departments has led to new collaborative work to promote safe driving in older adults, including a state funded intervention study.

